# Supplementary material for: Identification of photoperiod-induced specific miRNAs in the adrenal glands of Sunite sheep (Ovis aries)
Source: Front Vet Sci. 2022 Jul 22;9:888207. doi: 10.3389/fvets.2022.888207 (PMC9354845; doi:10.3389/fvets.2022.888207)
Supplement: Supplementary file 1 [file Data_Sheet_1.ZIP › Supplementary file/miRDeep2 file.zip!/miRDeep2 file/Novel_Structure/SP42A2/Novel_123.pdf]

Provisional ID	:	NC_019484.2_42784
Score total	:	16
Score for star read(s)	:	3.9
Score for read counts	:	8.8
Score for mfe	:	1.7
Score for randfold	:	1.6
Score for cons. seed	:	
Total read count	:	29
Mature read count	:	28
Loop read count	:	0
Star read count	:	1

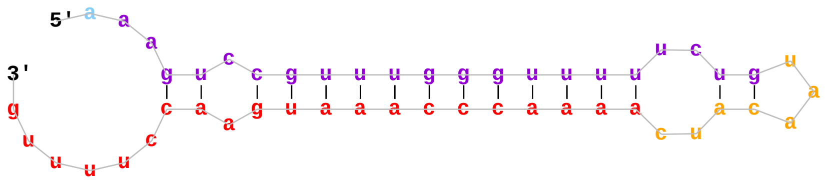

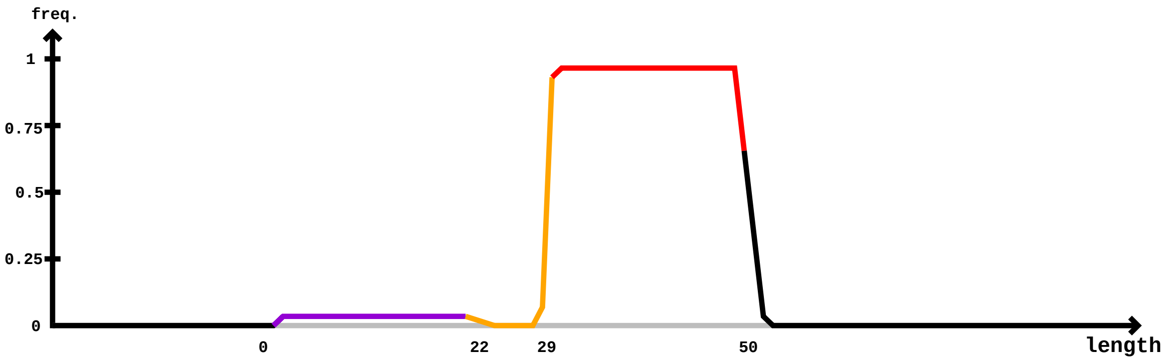


```
                         Star                        Mature
5'- uaaauauauuggguuggcccaaaaguccguuuggguuuuucuguaacaucaaaacccaaaugaaccuuuuggccaaccuaauaacuuauuuuguaauaacuuuaagugaag   -3'    obs
    uaaauauauuggguuggcccaaaaguccguuuggguuuuucuguaacaucaaaacccaaaugaaccuuuuggccaaccuaauaacuuauuuuguaauaacuuuaagugaag          exp
    ......(((((((((((((.(((((..((((((((((((..((...))..))))))))))))...))))))))))))))))))(((((....((....))..)))))....    reads    mm    sample
    ......................aaguUcguuuggguuuuucug....................................................................    1        1     seq
    .................................................caaaacccaaaugaacUuuuug........................................    1        1     seq
    .................................................Gaaaacccaaaugaaccuuuug........................................    1        1     seq
    ..................................................aaaacccaaaugaacUuuuu.........................................    9        1     seq
    ..................................................aaaacccaaaugaacUuuuug........................................    16       1     seq
    ...................................................aaacccaaaugaacUuuuuggc......................................    1        1     seq
```
